# Simultaneous Determination of Metal Ions in Zinc Sulfate Solution Using UV–Vis Spectrometry and SPSE-XGBoost Method

**DOI:** 10.3390/s20174936

**Published:** 2020-08-31

**Authors:** Fei Cheng, Chunhua Yang, Can Zhou, Lijuan Lan, Hongqiu Zhu, Yonggang Li

**Affiliations:** 1 School of Automation, Central South University, Changsha 410083, China; feicheng@csu.edu.cn (F.C.); ychh@csu.edu.cn (C.Y.); lijuan.lan@csu.edu.cn (L.L.); hqcsu@csu.edu.cn (H.Z.); liyonggang@csu.edu.cn (Y.L.); 2 State Key Laboratory of High Performance Complex Manufacturing, Changsha 410083, China

**Keywords:** zinc hydrometallurgy, metal ion measurement, UV–vis spectroscopy, feature selection and combination, singular perturbation spectrum estimator, extreme gradient boosting

## Abstract

Excessive discharge of heavy metal ions will aggravate environment pollution and threaten human health. Thus, it is of significance to real-time detect metal ions and control discharge in the metallurgical wastewater. We developed an accurate and rapid approach based on the singular perturbation spectrum estimator and extreme gradient boosting (SPSE-XGBoost) algorithms to simultaneously determine multi-metal ion concentrations by UV–vis spectrometry. In the approach, the spectral data is expanded by multi-order derivative preprocessing, and then, the sensitive feature bands in each spectrum are extracted by feature importance (VI score) ranking. Subsequently, the SPSE-XGBoost model are trained to combine multi-derivative features and to predict ion concentrations. The experimental results indicate that the developed “Expand-Extract-Combine” strategy can not only overcome problems of background noise and spectral overlapping but also mine the deeper spectrum information by integrating important features. Moreover, the SPSE-XGBoost strategy utilizes the selected feature subset instead of the full-spectrum for calculation, which effectively improves the computing speed. The comparisons of different data processing methods are conducted. It outcomes that the proposed strategy outperforms other routine methods and can profoundly determine the concentrations of zinc, copper, cobalt, and nickel with the lowest RMSE${}_{P}$. Therefore, our developed approach can be implemented as a promising mean for real-time and on-line determination of multi-metal ion concentrations in zinc hydrometallurgy.

## 1. Introduction

Zinc metal smelting wastewater contains multiple toxic metal ions, such as zinc, copper, cobalt, and nickel. Irrational discharge of heavy metal ions will cause serious harm to the ecological environment [1,2]. At present, the concentrations of metal ions are mostly acquired via off-line analysis in the laboratory, which is laborious, time-consuming, connects with many errors and chemical costs, and leads to blind control of wastewater discharge. Hence, the real-time and accurate detection of metal ions is urgently needed [3].

As for better online monitoring methods, optical detection methods are widely used because of its high efficiency and low laboriousness, such as ultraviolet-visible (UV–vis) spectroscopy [4,5], atomic absorption spectroscopy (AAS) [6], near-infrared spectroscopy (NIRS) [7], surface-enhanced raman spectroscopy (SERS) [8], laser-induced breakdown spectroscopy (LIBS) [9], and so on. Among these, the UV-vis spectrophotometry can achieve online analysis on multi-ions without expensive sample pretreatment and is easily operated, making it cheaper and faster in the applications [10,11,12]. Our previous work focused on detecting copper and cobalt concentrations using UV–vis spectroscopy and multivariate regression model based on the wavelet denoising and locally weighted partial least squares methods [13,14]. However, due to the complex background and similar chemical properties of detected ions, the spectra are excessively overlapped and exist severe nonlinearity. The denoising method and regression model based on full spectrum will become invalid. The characteristic information of each ion is difficult to distinguish and extract when the ion species increased. Moreover, the external environment unavoidably generates noise interference, resulting in inconsistent intensity of spectral signals. All these problems make it arduous for the spectral quantitative analysis of complex mixed solution and seriously restrict the application of spectral technology.

To establish a quantitative analysis model, the works of predecessors can be roughly divided into three parts: spectral preprocessing, feature selection, and multivariate calibration. For spectral preprocessing, the commonly used methods are denoising and derivatives. The derivative method can reconstruct the spectral peak and eliminate the background signal interference [15,16]. Moreover, the ability to distinguish subtle changes in similar spectra is considerably enhanced in the derivative spectrum [17]. However, most studies usually select a single derivative approach, which may not be sufficient for analysis of severe overlapped spectra. Li et al. proposed the singular perturbation spectral estimator (SPSE) based on the singular perturbation technique and Taylor series to obtain high quality derivative spectra from the measured spectrum with noise [18,19,20]. Since the obtained spectrum is relatively simple, lacks detail information, and contains a large amount of arbitrary noises, it is vital to adopt diversified preprocessing methods that provide abundant and accurate information.

Frequently-used feature selection methods in spectroscopy are uninformative variable elimination (UVE) [21] and competitive adaptive reweighted sampling (CARS) [22]. It is considered that variable combination has a great influence on prediction performance [23]. Even when the subsets containing less important variables are combined, they can achieve a good predictive performance [24,25]. Therefore, the idea of variable combination is introduced into the spectrum analysis. For multivariate calibration, the commonly applied approaches are the linear method (e.g., partial least squares (PLS)) and the nonlinear modeling method (e.g., support vector machine (SVM)) [26,27]. At present, ensemble learning becomes a common technology to enhance the generalization ability by combining the prediction results of multiple base learners [28,29,30]. Extreme gradient boosting (XGBoost) is an iconic ensemble learning algorithm proposed by Chen et al. [31]. XGBoost has many advantages in processing nonlinear data and can extract features from variables containing noise and redundant information. Numerous studies demonstrate that it has promoted prediction accuracy and performed remarkable results for spectral analyses in different domains [32,33,34]. However, there are still few works to incorporate this sophisticated strategy into spectral quantitative analysis of heavy metal ions in solution.

Motivated by the above factors, this article introduces SPSE and XGBoost into UV–vis spectrometry for the first time to measure multi-metal ion concentrations. In view of the redundant noise and intricate correlation, the SPSE is employed to expand the multi-order derivative spectra with high accuracy and strong resistance of disturbance. The ensemble XGBoost model is used to extract the feature variables and rank the importance score. The sensitive feature bands in each spectrum are integrated to form new characteristic variable sets and the ion concentrations are predicted. Afterwards, the multi-derivative feature subset combination is considered to further promote the prediction precision. Finally, to validate the performance of the “Expand-Extract-Combine” strategy in SPSE-XGBoost, the comprehensive analyses among CARS-PLS, UVE-LS-SVM, and XGBoost are carried out. The remainder of this article is organized in the following sections. Section 2 describes the experimental procedure, in which the basic concepts of SPSE and XGboost are given, respectively. Then the proposed modeling framework and procedure are introduced. In Section 3, the validation of comparative results and overall performance of each model are discussed. Conclusions are drawn in Section 4.

## 2. Materials and Methods

### 2.1. Experimental Apparatus and Samples

A T9 UV–vis spectrophotometer (Beijing Purkinje General Instrument Co., Ltd., Beijing, China) is used to measure the spectrum. The T9 spectrophotometer utilizes a high-performance xenon lamp and double beam optical system, which can achieve spectral scanning over a wide wavelength range of 185 nm to 900 nm. A computer (Lenovo Group, Beijing, China) receives the spectral data via a UV-Win Software (Beijing Purkinje General Instrument Co., Ltd., Beijing, China). UV-Win software provides complete instrument control and a set of mathematical tools to analyze the measurement results.

The main metal ions in the hydrometallurgy wastewater of Zhuzhou Smelter Company are Zn(II), Cu(II), Co(II), and Ni(II), in which the concentration of Zn(II) is 20–250 times that of the other metal ions. Huge difference of ion concentrations can lead to inconsistent intensity of spectral and severe masking problems. Hence, it is extremely significant to select appropriate experimental reagents to ensure the precision of simultaneous determination for the multiple ions. The reagents and their optimized dosage are as follows: 0.4% Nitroso R salt chromogenic agent solution: 2.5 mL; HAc-NaAc buffer solution: pH = 5.5, 5 mL. The concentrations of zinc standard solutions are 1 g/L. Copper, cobalt, and nickel standard solutions are all 12.5 mg/L. All reagents are of analytical grade and added in a 25 mL colorimetric tube. The specific operation procedure of the experiment is as follows: In the 25 mL colorimetric tube, add 5.0 mL HAc-NaAc buffer solution. Add zinc standard solution and proper amount of copper, cobalt, nickel ion standard solution. Add 2.5 mL 0.40% Nitroso R salt chromogenic solution, shake well to make the chromogenic reaction fully react. Add distilled water to make the volume up to 25 mL. Prepare reagent blank. Adjust the instrument to zero. Place the sample in a 1 cm quartz cuvette, and use the reagent blank as a reference.

**Figure 1:**
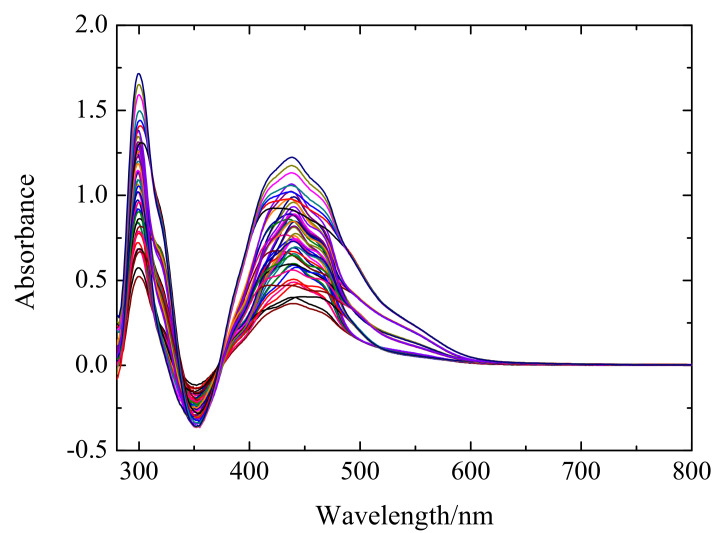
The original absorption spectrum of 49 samples for training and testing.

**Figure 2:**
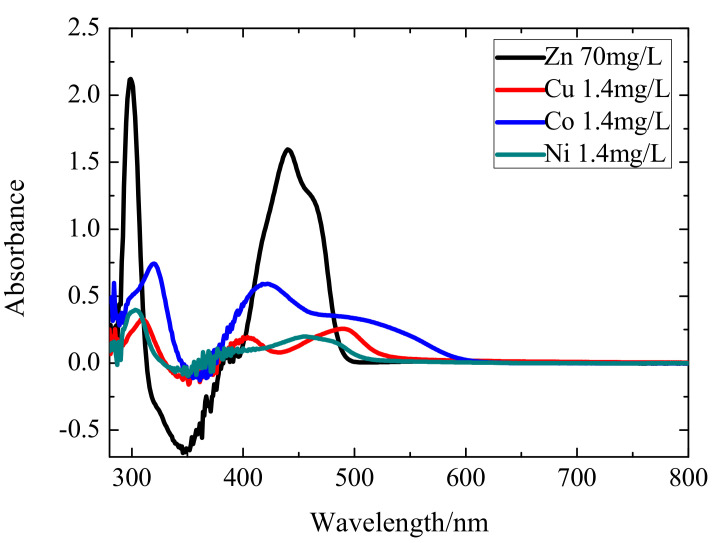
The single ion absorption spectrum for zinc, copper, cobalt, and nickel, respectively.

In this study, 49 groups of mixed solutions are analyzed. Figure 1 shows the original spectra of the 49 mixed ion solutions. The measured UV–vis spectrum wavelength ranges from 280 nm to 800 nm with 1 nm scanning resolution. Each sample is scanned three times and the averaged spectrum is obtained for calculation. Among them, the concentration range of Zn(II) is between 10 mg/L and 70 mg/L and the concentrations of Cu(II), Co(II), and Ni(II) all range from 0.2 mg/L to 1.4 mg/L. The concentration of Zn(II), Cu(II), Co(II), and Ni(II) in the solutions are shown in Table 1. Figure 2 is the spectra of single ion solution of Zn(II), Cu(II), Co(II), and Ni(II), which exhibits that the peaks of ions are adjacent to each other. This is because the competitive reaction between Zn(II) and other impurity metal ions aggravates the spectrum overlapping and masking. Meanwhile, the peak shape and movement tendency of ions are similar due to their resemble chemical properties. Besides, a negative peak occurs mainly because the absorbance value of Zn(II) complex in the measured solution is less than of the reagent blank at 320–380 nm. A large amount of noise appear at 280–300 nm and 300–400 nm mainly because of the high absorbance of the reference solution, making the spectrum nonlinear at these ranges. Therefore, the traditional calibration method based on the full spectrum can hardly achieve a high detection accuracy.

### 2.2. Multi-Derivative Spectral Reconstruction by SPSE

Due to the random noise and overlapping problems in the original spectra of complex mixtures, the derivative spectra method is widely used in spectral analysis of multicomponent calibration. To decrease the background error and separate the overlapping absorption band, the singular perturbation spectrum estimator (SPSE) [19,20] is applied. The estimator is based on inverse Taylor series, which takes the advantage of scale separation to obtain the simplified original problems [35].

Assuming that the spectral signal $u(v_{1})$ is given at any wavelength $v_{1}$ and that $u(v_{1})$ is differentiable k+1 times at any $v_{1}$. Define $v_{1}=v+\varepsilon$, where ε is the system perturbation parameter and sufficiently small. For higher-order derivative estimators, because $\lim_{\varepsilon \to 0}\;x_{i}=u^{\left(i-1\right)}\left(v\right)\left(i=1,2,\cdots ,n\right)$, the Taylor series of spectral signal can be approximated as linear differential system and the description of SPSE is obtained as follows:

(1) $$\left\{\begin{array}{c} {\dot{x}}_{1}\left(v\right)=x_{2}\left(v\right)\\ {\dot{x}}_{2}\left(v\right)=x_{3}\left(v\right)\\ {\dot{x}}_{3}\left(v\right)=-\frac{6}{\varepsilon^{3}}\left(x_{1}\left(v\right)-\hat{u}\left(v\right)\right)-\frac{6}{\varepsilon^{2}}x_{2}\left(v\right)-\frac{3}{\varepsilon }x_{3}\left(v\right)\\ y\left(v\right)=x_{1}\left(v\right), \end{array}\right.$$

where $\hat{u}\left(v\right)=u\left(v_{1}\right)$ is the measured spectral signal; $\left({\dot{x}}_{1},{\dot{x}}_{2},{\dot{x}}_{3}\right)$ are the state items of the differentiator; $x_{1}\left(v\right)$ is the denoising spectrum of the measured signal u(v); and $x_{2}\left(v\right)$ and $x_{3}\left(v\right)$ are the first-order and second-order derivative spectrum, respectively.

Since the SPSE only has the perturbation parameter ε to adjust, it can overcome the restriction of inconsistent parameter selection. Meanwhile, a large amount of additive noise is eliminated by the successive multiple integral parts. Thus, it is concluded that the denoising spectrum and multi-order derivative spectrum can be estimated, effectively suppressing random noise and redundant background signals in the spectrum.

### 2.3. eXtreme Gradient Boosting Based on Feature Importance Ranking

**Figure 3:**
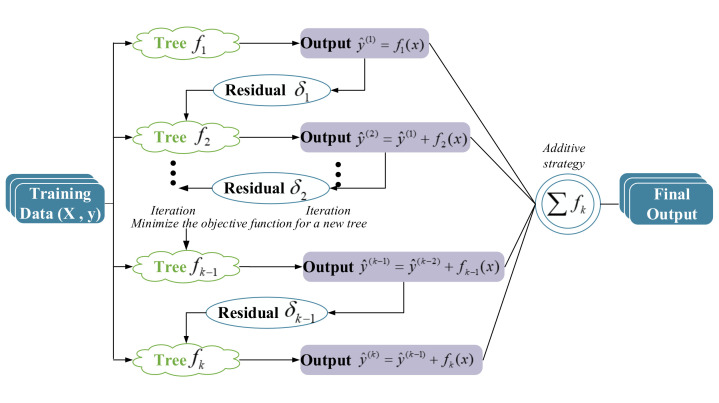
The structure of extreme gradient boosting.

Extreme gradient boosting (XGBoost) [31] is a novel tree learning algorithm which achieves considerable result for sparse data processing. It takes classification and regression tree (CART) as the base learner. Figure 3 illustrates the basic structure of XGBoost, in which *X* is the spectral absorbance matrix in this model and *y* is the concentration of a certain metal ion. According to the additive training strategy of boosting, each tree is constructed based on learning from the residual δ of the previous tree. ${{\hat{y}}_{i}}^{\left(k\right)}={{\hat{y}}_{i}}^{\left(k-1\right)}+f_{k}\left(x_{i}\right)$ is the prediction of the *k*-th iteration. At every iteration, XGboost optimizes the model and decreases the prediction error. The final prediction output ${\hat{y}}_{i}$ is generated by the weighted summation of trees as follows:

(2) $${\hat{y}}_{i}=\sum_{k=1}^{K}f_{k}\left(x_{i}\right),f_{k}\in F,$$

where F is the space of functions containing all regression trees; *K* denotes the number of trees. To learn function $f_{k}$ of each tree, XGBoost establishes an objective function with regularization:

(3) $$L\left(\phi \right)=\sum_{i}l(y_{i},{\hat{y}}_{i})+\sum_{k}\Omega (f_{k}),$$

where ϕ is all learnable parameters in XGBoost; $l(y_{i},{\hat{y}}_{i})$ is the loss function representing the error between the predicted concentration ${\hat{y}}_{i}$ and the actual concentration $y_{i}$, the smaller the *l* is, the better the performance of the algorithm; $\Omega (f_{k})$ is the regularization term to penalize the model complexity and prevent over-fitting. When XGBoost uses the square loss function to measure error, the second derivative Taylor expansion of the loss function can assist the model to optimize the objective quickly. The second derivative Taylor expansion of the loss function after *k*-th iteration is given as follows:

(4) $$L{\left(\phi \right)}^{\left(t\right)}≃\sum \left[l\left(y_{i}^{\left(t\right)},{\hat{y}}_{i}^{\left(t-1\right)}\right)+g_{i}f_{t}\left(x_{i}\right)+\frac{1}{2}h_{i}f_{t}^{2}\left(x_{i}\right)\right]+\Omega \left(f_{t}\right),$$

where $g_{i}$ and $h_{i}$ are the first and second derivative of the loss function. It can be learned that the loss function only depends on the first and second derivatives of each data point. To predict the ion concentrations, the essential step in the XGBoost learning algorithm is to optimize the XGBoost algorithm parameters, booster parameters, and learning parameters.

Additive tree boosting model enables XGBoost to flexibly use variables in different areas of the output space. This model can perform effective feature selection and capture higher-order interactions. Thus, XGBoost in this paper is used not only for feature selection but also for prediction. After all boosting trees are built, XGBoost can calculate out the importance of each feature. XGBoost generates the ranking of all features based on variable importance (VI). The VI score measures the frequency of individual feature that is used to build trees. The more times a feature is selected for splitting trees, the more valuable it proves to be in the model. In this paper, the feature importance ranking of VI score is regarded as the basis of feature selection.

### 2.4. The Proposed SPSE-XGBoost Approach

**Figure 4:**
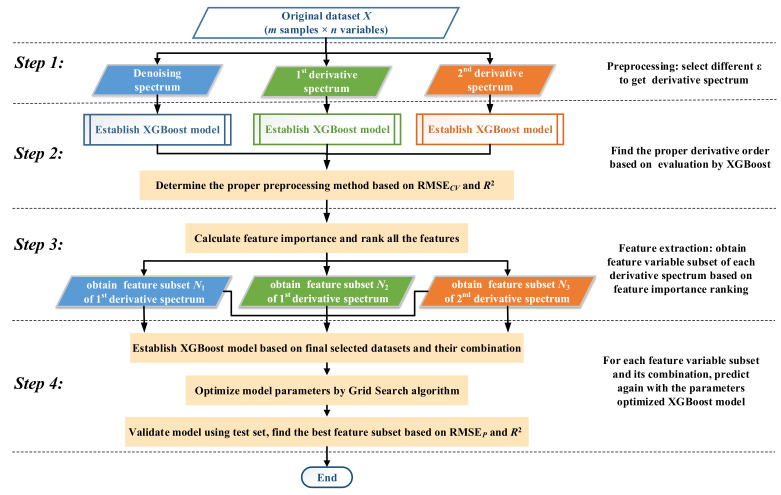
Flow chart of the proposed singular perturbation spectral estimator (SPSE)-XGBoost model.

In view of the merits of SPSE and XGBoost, in this paper, they are integrated to establish a novel calibration model, shorted as SPSE-XGBoost. The focus of this approach is to explore the benefits of combining feature subsets of multi-order derivative spectrum and, meanwhile, introduce the ensemble XGBoost algorithm as key model in feature selection and prediction of metal ion concentrations such as zinc, copper, cobalt, and nickel. To assess the quality of SPSE-XGBoost model, the root mean square error (RMSE), the coefficient of determination ($R^{2}$), the mean absolute percentage error (MAPE), and the maximum absolute percentage error (MaxAPE) are utilized as the main evaluation criteria in the proposed approach. Smaller RMSE, MAPE, and MaxAPE represent better model precision. Figure 4 illustrates the flow chart of the proposed SPSE-XGBoost model that is comprised of the following four steps.

**Step** **1**  The samples are divided into training set and test set. The *X* matrix contains all variables of training set. Then, select different singularly perturbation parameter ε of SPSE to obtain the denoising spectrum and the first-order and second-order derivative spectra.
**Step** **2**  Perform XGBoost modeling with cross validation for each spectrum. Since different derivative spectra have different predictive capability, the results are further compared and analyzed to select preferable derivative order for each ion.
**Step** **3**  Calculate the VI score and rank to extract features. The sensitive feature in each spectrum are integrated to form new feature subsets.
**Step** **4**  Combine the feature subsets and build SPSE-XGBoost model to predict ions concentration in the test set. Determine the optimum variables combination via RMSE${}_{P}$ and $R^{2}$.

In brief, the proposed method aims to find the best subset of features for multi-metal ions prediction and analyze the effects of variable selection and combination by an “Expand-Extract-Combine” strategy. “Expand” refers to the derivative preprocessing procedure that expands the spectral space of original data. “Extract” means that individual variable is ranked and selected by the VI score and “Combine” defines that multi-derivative feature subset combination is considered to promote prediction performance.

## 3. Results and Discussion

### 3.1. Multi-Order Derivative Reconstruction Pretreatment

**Figure 5:**
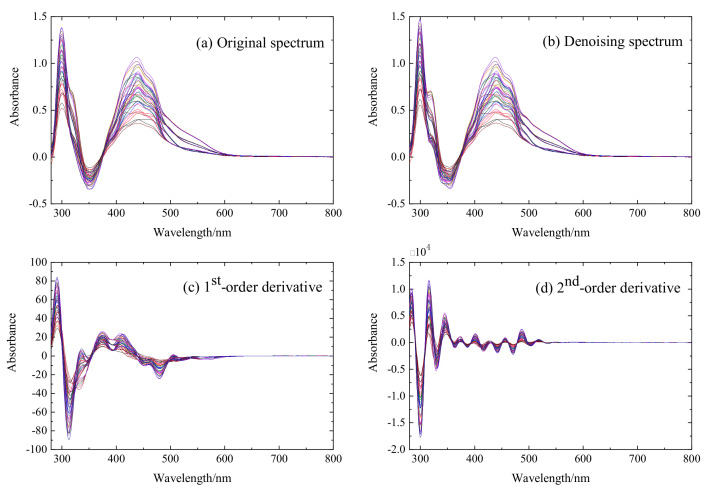
The original, denoising, first-order, and second-order derivative spectra of the training set.

The samples were firstly divided into a training set (39 samples) and a test set (10 samples) using the Kennard-Stone algorithm [36]. To highlight the characteristic information of each ion, different singularly perturbation parameter ε is selected in the SPSE to get the denoising spectrum and the first-order and second-order derivative spectra. To obtain the best preprocessing results, the ε in our work were set as 0.007 for denoising spectrum, 0.013 for first-order spectrum, and 0.016 for second-order spectrum, respectively. It can be seen from Figure 5 that subtle changes in the original spectrum is obviously reinforced after pretreatment, highlighting the ionic difference. The denoising spectrum does not make significant difference with the original spectrum in the shape. The absorbance and resolution are greatly enhanced in the derivative spectra, which reasonably expands the data space and thus provides abundant features for variables selection.

To ensure the reliability of preprocessing, the prediction results of XGBoost model with 10-fold cross-validation using full-spectrum (280–800 nm) is preliminarily analyzed as shown in Table 1. The RMSE${}_{CV}$ and $R^{2}$ are used to evaluate the predictive ability of the model. The MAPE and MaxAPE are also calculated. Table 2 reveals that different derivative spectrum models provide different prediction results. For zinc, the accuracy of models established by the first-order and second-order spectrum are improved compared with the original spectrum. Although the noise signal is suppressed in the denoising spectrum, some important characteristics of zinc may be inevitably weakened, so the predicting result of the denoising spectrum is the worst. For copper, most of the original signals are masked by zinc, so the denoising spectrum and the first-order derivative spectrum have the strongest prediction ability, and their MAPE are greatly reduced. Whereas for cobalt, only the model established by the second-order derivative spectrum has a impressive accuracy. By calculating the variation in absorbance change rate, spectral peaks become exceedingly sharp and the overlapping spectral bands are in a way separated. Therefore, the characteristic information of cobalt stands out conspicuously. For nickel, the model with the denoising spectrum and the first-order derivative is preferable. The second-order derivative model is the worst. This is because the signal of nickel is the weakest in zinc sulfate solution. The second-order derivative spectrum inevitably amplifies the instability of signal and reduces the prediction precision.

Overall, we have three interesting findings: (i) Different preprocessing method has different effects on the prediction ability of different ions in the same solution. For example, the denoising spectrum has a positive effect on copper, cobalt, and nickel but negative on zinc; (ii) derivative pretreatment provides more abundant and effective data for spectral prediction, which can improve the accuracy of model; (iii) a single derivative full-spectrum cannot meet the industrial requirements that the average error of the measurement of should not exceed 5%, and the maximum error should not exceed 10%. Therefore, the derivative variables extraction and combination will be considered to maximize the effective information in the following subsections.

### 3.2. Variable Selection and Feature Importance Analysis

After preprocessing, we obtain the high-dimension spectral data. Before modeling the spectral data with small sample yet high dimensions, it is extremely necessary to lessen dimension with appropriate variable selection method. For VI score ranking, XGBoost extracts feature variables by calculating the importance ranking of all variables. The times of a feature selected as a splitting tree node are regarded as the VI scores to measure the feature importance.

**Figure 6:**
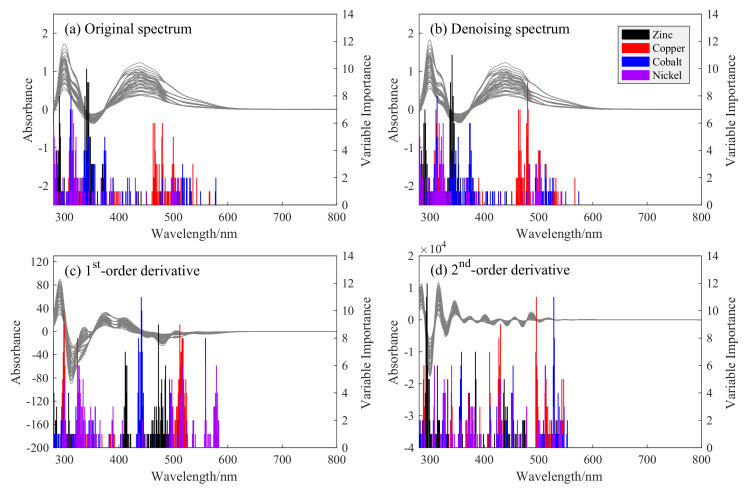
Characteristic variable selection and feature importance (VI) score results of zinc, copper, cobalt, and nickel ions in the original (**a**), denoising (**b**), first-order (**c**), and second-order derivative (**d**) spectra. The gray curves in each subplot are the spectra of the training set (Figure 6).

The variables selection for multi-metal ions and their VI scores of different spectra are shown in Figure 6. From Figure 6a,b, we can find that the original and denoising spectra have approximately the same range of wavelength bands, indicating that the denoising pretreatment does not change feature position in the selected variables. The selected variables of zinc are mainly located in the range of 280–380 nm. Copper has a strong concentrated feature band located at 460–510 nm. This is consistent with the characteristic peak at 490 nm in the single ion spectrum of copper, which can be clearly seen at Figure 2. In the original and denoising spectra, the cobalt ions are distributed in a dispersed manner, ranging from 280 nm to 580 nm. Nickel ions are mainly distributed around 280–400 nm and 500 nm, which is related to the three characteristic peaks of nickel ions at 310 nm, 410 nm, and 500 nm in the single ion spectrum. In general, the VI scores of the denoising spectrum is higher than that of the original spectrum, especially for copper and nickel, which reflects that the elimination of random noise is conducive to feature selection and information mining.

When taking the first-order and second-order derivations, the selected variables are distributed to a wide range and move backwards. The selected feature bands of the different ions become distinguished and more concentrated, suggesting that the derivations can effectively expand the spectral data space for feature selection and separate overlapping spectral peaks of ions. For example, in the first-order derivative spectrum, the characteristic regions of zinc move to 400–420 nm and 450–500 nm, while the variables of copper, cobalt, and nickel are relocated at 500–520 nm, 430–450 nm, and 550–580 nm, respectively. These characteristic bands are all independent of each other and skillfully avoid the high noise bands at 280–380 nm. In particular, the variable selection of the first-order derivative spectrum is more concentrated, and the second derivative has a scattered distribution before 600 nm due to the large number of characteristic peaks and narrow peak shape.

The number of the selected characteristic variables of the corresponding metal ions under different preprocessing methods is listed in Table 3. Compared with the full-spectrum prediction, the calculated variable for one ion in the XGBoost model is reduced to below 87. Thus, using the selected feature subset can not only highlight the characteristic information for a specific metal ion but also remarkably improve the calculation speed of the model.

### 3.3. XGBoost Model with Variable Combination

To meliorate the prediction ability of the model, this approach also adopts the variable combination strategy. Variable combination takes advantage of the diversity of feature variables. It is also worth mentioning that to our best knowledge, the effect of variable combination is not considered in any existing method to predict the metal ion concentrations. Hence, the selected subset of derivative variables and their combinations are applied to retrain the XGBoost model, respectively. The prediction results are compared and evaluated by the MAPE, MaxAPE, RMSE${}_{P}$, $R^{2}$ in Table 4. The optimal model usually exhibits the largest $R^{2}$ and the lowest RMSE${}_{P}$ value. To meet the needs of industrial on-line detection, MAPE and MaxAPE are also required to be under 10%.

According to model results, it can be observed that the modeling outcome of selected variable subsets is superior to that based on the whole spectrum variables (Table 2). More interestingly, through the combination of subsets of characteristic region in the denoising and multi-order derivation spectra, the prediction ability of ions is further optimized. It is apparent from Table 4 that the RMSE${}_{P}$ and MAPE are effectively reduced. The MAPE of zinc, copper, cobalt, and nickel are 4.098%, 3.515%, 3.083%, and 4.331%, respectively. The MaxAPE of the four ions is less than 8.323%. Hence, it concludes that feature variable subset combination can make better use of the richness and diversity of the multi-order derivative spectra and contain characteristic variables to the greatest extent. Moreover, it further proves that even when some of the less important variables are combined, they can achieve good predictive performance. Especially for nickel with the strongest instability and the weakest absorbance, the MaxAPE and MAPE of the model prediction are greatly reduced, which meet the industrial requirements of accuracy.

**Figure 7:**
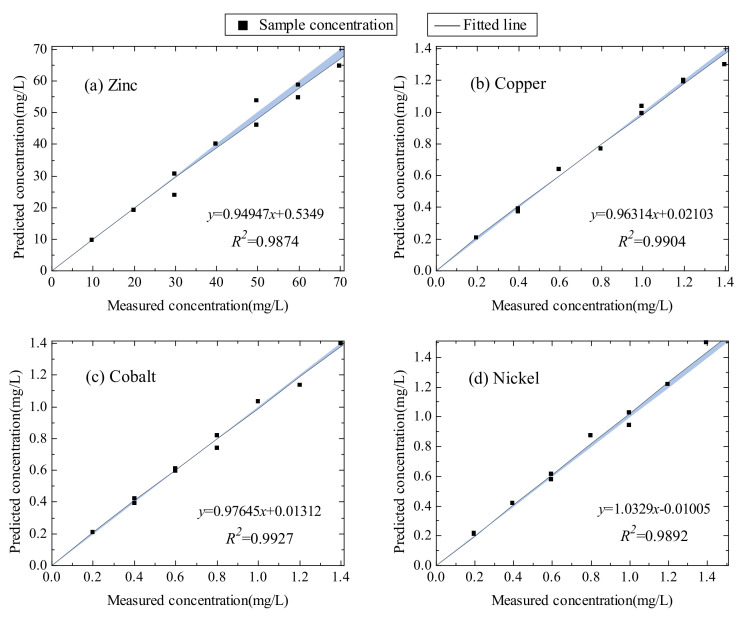
The fitting curves of zinc (**a**), copper (**b**), cobalt (**c**), and nickel (**d**) ions in the test set (10 samples).

The scatter points in Figure 7 illustrate the comparison of actual concentration and predicted concentration in the test set. The black lines are the fitted lines of actual and predicted concentration scatter points. The blue shaded areas stand for the gap between the 1:1 lines and the fitted lines. When the fitted line is closer to the 1:1 line, the blue shaded area is smaller, indicating a better correlation between the prediction result and actual concentration. As exhibited, the fitted lines of each ion closely approach to the 1:1 lines and the $R^{2}$ values of each ion are higher than 0.987, suggesting that the proposed method has favorable accuracy and promising effect in detecting the concentrations of zinc, copper, cobalt, and nickel ions in zinc sulfate solution.

**Figure 8:**
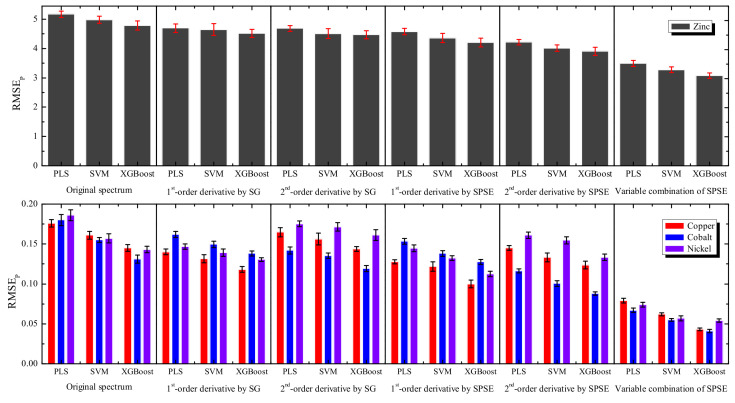
Comparison results among different preprocessing and modeling methods for zinc, copper, cobalt, and nickel ions.

To further verify the performance of the proposed model, different derivatives pretreatment methods, such as Savitzky-Golay (SG) derivative algorithm and traditional modeling methods (CARS-PLS, UVE-LS-SVM), are also carried out and compared in Figure 8. Note that CARS-PLS and UVE-LS-SVM are shortened as PLS and SVM in the figure, respectively. All the methods are conducted 10 times to obtain the statistical results. Only the variable combination method of SPSE uses subset combination of characteristic variables, while the other methods are all based on the full spectral variables. From Figure 8, the performances of the different algorithms are distinct. For the single preprocessing methods, the original spectral data almost have the highest RMSE${}_{P}$ due to the noise and masking problems in the raw spectrum. The exceptions are the first-order derivative for cobalt and the second-order derivative for nickel, which have been interpreted in Section 3.1. The derivative spectra using SPSE always achieve lower RMSE${}_{P}$ than the corresponding SG, indicating that SPSE is superior to SG. For the modeling methods, the XGBoost outperforms the other two traditional calibration methods as shown in the figure. It is worth mentioning that the combination of variables has a significant effect on model promotion and yields the lowest RMSE${}_{P}$, signifying the best prediction ability of SPSE-XGBoost in general.

All in all, it is easily concluded that the SPSE and XGBoost algorithms have better capabilities in expanding the spectral information, extracting the effective variables, and predicting ion concentrations in the UV-vis spectrum analysis. When all of the feature subsets from the derivative spectra are combined, the developed SPSE-XGBoost method can further enhance the measurement accuracy. Therefore, our proposed “Expand-Extract-Combine” strategy of SPSE-XGBoost in this article has a great potential for the real-time and on-line detection of multi-metal ion concentrations in hydrometallurgy wastewater.

## 4. Conclusions

In this work, we developed the SPSE-XGBoost approach to simultaneously measure the multi-metal ion concentrations by UV–vis spectroscopy in the complex zinc sulfate solutions. At first, the spectral data was expanded by the denoising and multi-order derivative preprocessing in SPSE. Then, the feature variables were extracted by accounting the VI score ranking using the ensemble XGBoost algorithm. Finally, the feature subsets from the derivative spectra were combined to further promote the accuracy in determining zinc, copper, cobalt, and nickel ion concentrations. The adequate analyses indicate that the “Expand-Extract-Combine” strategy in the SPSE-XGBoost approach has the properties of suppressing the redundant noises, extending the spectral feature data to a broad space, extracting the spectral feature band for a specific metal ion, improving the computing speed, and obtaining the high-precise results, and so on. The comparisons with the conventional SG preprocessing, CARS-PLS, and UVE-LS-SVM methods illustrate the superior performances of our proposed method. With such analyses, our developed approach was proven to be suitable for real-time and on-line detection of multi-metal ion concentrations in hydrometallurgy wastewater.

## Figures and Tables

**Table 1:** The concentration of Zn(II), Cu(II), Co(II), and Ni(II) of 49 samples (mg/L).

NO.	Zn(II)	Cu(II)	Co(II)	Ni(II)	NO.	Zn(II)	Cu(II)	Co(II)	Ni(II)
1	10	0.2	0.4	0.6	26	40	1.0	0.6	0.2
2	10	0.4	0.8	1.2	27	40	1.2	1.0	0.8
3	10	0.6	1.2	0.4	28	40	1.4	1.4	1.4
4	10	0.8	0.2	1.0	29	50	0.2	0.4	0.6
5	10	1.0	0.6	0.2	30	50	0.4	0.8	1.2
6	10	1.2	1.0	0.8	31	50	0.6	1.2	0.4
7	10	1.4	1.4	1.4	32	50	0.8	0.2	1.0
8	20	0.2	0.4	0.6	33	50	1.0	0.6	0.2
9	20	0.4	0.8	1.2	34	50	1.2	1.0	0.8
10	20	0.6	1.2	0.4	35	50	1.4	1.4	1.4
11	20	0.8	0.2	1.0	36	60	0.2	0.4	0.6
12	20	1.0	0.6	0.2	37	60	0.4	0.8	1.2
13	20	1.2	1.0	0.8	38	60	0.6	1.2	0.4
14	20	1.4	1.4	1.4	39	60	0.8	0.2	1.0
15	30	0.2	0.4	0.6	40	60	1.0	0.6	0.2
16	30	0.4	0.8	1.2	41	60	1.2	1.0	0.8
17	30	0.6	1.2	0.4	42	60	1.4	1.4	1.4
18	30	0.8	0.2	1.0	43	70	0.2	0.4	0.6
19	30	1.0	0.6	0.2	44	70	0.4	0.8	1.2
20	30	1.2	1.0	0.8	45	70	0.6	1.2	0.4
21	30	1.4	1.4	1.4	46	70	0.8	0.2	1.0
22	40	0.2	0.4	0.6	47	70	1.0	0.6	0.2
23	40	0.4	0.8	1.2	48	70	1.2	1.0	0.8
24	40	0.6	1.2	0.4	49	70	1.4	1.4	1.4
25	40	0.8	0.2	1.0					

**Table 2:** Error evaluations of full-spectrum among different preprocessing methods for metal ions concentration prediction.

	Preprocessing	MAPE(%)	MaxAPE(%)	RMSE${}_{\mathrm{CV}}$(mg/L)	$R^{2}$
	Raw	13.777	21.534	4.785	0.792
**Zinc**	Denoising	16.718	24.621	4.947	0.749
	**1st derivative**	**8.744**	**13.220**	**4.242**	**0.892**
	**2nd derivative**	**8.235**	**11.395**	**3.941**	**0.921**
	Raw	13.762	18.781	0.145	0.746
**Copper**	**Denoising**	**8.023**	**12.789**	**0.115**	**0.924**
	**1st derivative**	**7.236**	**11.604**	**0.105**	**0.938**
	2nd derivative	11.819	14.355	0.139	0.824
	Raw	11.213	23.958	0.131	0.773
**Cobalt**	Denoising	11.051	21.545	0.146	0.737
	1st derivative	10.792	17.897	0.134	0.756
	**2nd derivative**	**6.254**	**12.032**	**0.099**	**0.901**
	Raw	12.463	23.057	0.143	0.779
**Nickel**	**Denoising**	**8.529**	**14.573**	**0.114**	**0.907**
	**1st derivative**	**9.322**	**15.496**	**0.118**	**0.894**
	2nd derivative	17.758	25.619	0.150	0.738

**Table 3:** The number of selected characteristic variables by XGBoost of zinc, copper, cobalt, and nickel ions under different preprocessing methods.

Preprocessing	Number of Characteristic Variables			
	Zinc	Copper	Cobalt	Nickel
Raw	57	77	78	68
Denoising	62	83	82	71
1st derivative	63	86	73	72
2nd derivative	59	75	81	87

**Table 4:** Model prediction results of extracted variables subsets and their combinations under different preprocessing methods for metal ion concentration prediction.

	Feature Subset	MAPE(%)	MaxAPE(%)	RMSE${}_{P}$(mg/L)	$R^{2}$
	Denoising	∖	∖	∖	∖
**Zinc**	1st derivative	6.942	9.433	3.569	0.948
	2nd derivative	6.271	9.185	3.412	0.956
	**Combination**	**4.098**	**7.986**	**3.107**	**0.987**
	Denoising	4.241	9.452	0.056	0.977
**Copper**	1st derivative	3.924	8.404	0.051	0.984
	2nd derivative	∖	∖	∖	∖
	**Combination**	**3.515**	**6.939**	**0.043**	**0.990**
	Denoising	∖	∖	∖	∖
**Cobalt**	1st derivative	∖	∖	∖	∖
	**2nd derivative**	**3.083**	**7.414**	**0.041**	**0.993**
	Combination	∖	∖	∖	∖
	Denoising	6.879	9.922	0.078	0.946
**Nickel**	1st derivative	4.612	9.067	0.072	0.953
	2nd derivative	∖	∖	∖	∖
	**Combination**	**4.331**	**8.323**	**0.054**	**0.989**
